# Short and mid-term neonatal outcomes in high-risk infants undergoing FICare: a case control study

**DOI:** 10.1038/s41390-024-03307-z

**Published:** 2024-06-10

**Authors:** Bárbara Moreno-Sanz, Marta Antón, María Teresa Montes, Marta Cabrera-Lafuente, Itsaso Losantos-García, Adelina Pellicer

**Affiliations:** 1 Department of Neonatology, La Paz University Hospital, Madrid, Spain; 2 Hospital La Paz Institute for Health Research-IdIPAZ, Madrid, Spain; 3 Biostatistics Department, Hospital La Paz Institute for Health Research-IdIPAZ, Madrid, Spain

## Abstract

**Background:**

FICare model has been evaluated mostly on the stable preterm infant.We have scaled the model to two implementation levels(basic/advanced),making it suitable for all high-risk neonates.We report on the short- and mid-term outcomes of infants enrolled in a pilot on FICare implementation at our NICU.

**Methods:**

During 52 months study period,families were invited to join the program if their newborns’ admission required neonatal specialized care for at least 3 weeks,and trained according to the program’s curricula.Following a rigorous sequential admission order,each case(FICare group:134 < 34 weeks;52 term newborns)was matched by a contemporary control(CC:134 < 34 weeks;52 term newborns)and 2 historical controls born within the 3 years prior to FICare site implementation(HC:268 < 34 weeks;104 term newborns),cared as usual

**Results:**

FICare intervention started by the end of first week of postnatal life.Rates of breastfeeding during admission and at discharge,and direct breastfeeding upon discharge were higher in FICare compared to CC and HC.Duration of intermediate care hospitalization(preterm and term cohorts)and total hospital length of stay (term cohorts)were shorter in FICare group.Use of Emergency Services after discharge was also lower in the FICare group

**Conclusions:**

Short and mid-term efficacy of FICare on health outcomes and family empowerment in a broader and highly-vulnerable neonatal population supports its generalization in complex healthcare neonatal services.

**Impact statement:**

- Scaling the FICare model to the critically ill, unstable premature and term infant is feasible and safe.
- The early intervention shows similar benefits in the short- and mid-term infants’ outcomes in the whole spectrum of neonatal specialized care.

## Introduction

Family Integrated Care (FICare) model includes parents as part of the health team in the process of caring for their own child. Thus, parents become the main caregivers of their child, being accompanied by the healthcare professionals. Since the start of the model’s implementation the focus has almost universally been the stable preterm infant who had no or low level respiratory support.^1–6^ The rationale of reducing the stress and anxiety in families and improving their empowerment to behave as parents was behind. Up to now, this model of care has demonstrated numerous benefits that include increased breastfeeding rates and weight gain,^1–3,7,8^ earlier exclusive enteral and oral nutrition,^5,7^ decreased nosocomial infection,^3,4,8^ shorter duration of supplemental oxygen and mechanical ventilation,^3,5,7,9^ or shorter length of hospital stay.^3,5,6,9^ Although most of the studies were designed to improve short-term results, some of them have also reported on the long-term benefits in neurobehaviour at 18 months, consisting on lower dysregulation scores indicating better self-regulation skills,^10^ and higher motor scores assessed by the Bayley-III Motor Scales.^11^ A lower risk of communication delays has also been reported associated to the FICare model.^12^

We have recently described the development and effort taken to successfully adapt and implement the FICare policies to make it suitable also in the unstable, extremely preterm infant as well as in other high-risk neonates suffering complex medical or surgical conditions.^13^ To do this, we scaled the model to two levels of implementation (basic and advanced) that started earlier than previously reported during the infants’ clinical course. Through a structured and individualized training program we succeeded to make FICare suitable for the entire spectrum of care of the high-risk neonate.^13^

The purpose of this report is to describe the short- (at term-equivalent age or hospital discharge, whatever came first) and mid-term (first 6 months from discharge) outcomes of patients who were enrolled in a pilot study on FICare implementation. The intervention was delivered in an open-bay facilities, so that to control for a possible contamination effect of the intervention, this case-control study includes, in addition to a contemporary non-FICare group(CC) attended according to standard policies, a historical cohort(HC). We hypothesized that our site-tailored FICare model is superior to standard NICU care delivery with regards to short-term health outcomes in high-risk newborns with prolonged hospital stay.

## Methods

### Study design

The study was carried out at the Department of Neonatology at La Paz University Hospital in Madrid, Spain, between July 2018 and October 2022. Each infant in the FICare group was matched (1:1) by the next patient of similar clinical characteristics immediately admitted to the NICU cared as usual (CC); and by 2 infants of a retrospective cohort (HC) admitted within the 3-years period prior to FICare implementation (1:2), following a rigorous sequential admission order, taking a wash up period of 3 months (January 2015 to April 2018). The study was approved by the Research Ethics Committee at La Paz University Hospital.

### Study participants and procedures

Both infant’s and family’s entry criteria should be fulfilled to be considered eligible. Potential candidates were preterm (birth weight ≤1500 g or gestational age ≤ 34 weeks) and term newborns admitted to NICU due to immaturity-related issues or any other peri-neonatal condition for whom a length of stay of at least 3 consecutive weeks was expected, with a decision to provide full life support. Infants were not eligible in case of critical illness unlikely to survive or were scheduled for early transfer to another hospital.

Regarding family caregivers, willingness to spend at least 6 h per day at NICU attending educational sessions and having an active involvement in care, in addition to no intellectual or language barriers to understanding were compulsory requirements. The intervention was not offered in the case of intellectual handicaps, psychiatric problems or under legal supervision, or language comprehension difficulties. In all participating infant-family dyads the informed consent was signed.

The project leader (AP) created a FICare implementation team formed by members of the local associations of veteran parents and a variety of NICU healthcare professionals, who carried out an analysis of current procedures for critical care to identify needs, wishes, and requirements. As a result of the analysis the following site-adapted FICare pillars were defined: the educational curricula for staff and family caregivers, the training and accreditation procedures, the specifications about tools and training materials, as well as the psychological support and physical facilities to be offered to ensure needs and challenges were properly covered. Immediately after enrolment, families received all the training materials as well as an individualized training schedule. The family training was carried out in a progressive and individualized manner, taking into account the infant’s clinical status and parental wishes and expectations. The complexity of care or the severity of disease state did not have an impact on the time to start the intervention that relayed on three cornerstones: cot-side face-to-face individualized theoretical and practical sessions by tasks, interactive workshops covering topics of general interest, and rigorous registry of teaching activities and task certifications in the corresponding logbook. Of note, the observed time to reach proficiency by task was within the expected time in 70% of the program contents. Detailed information about training methods and materials can be found elsewhere.^13^

Care was provided according to general NICU standards along the two study periods in the case of the HC and the CC, that did not significantly varied. Basically, the unit was a 24/7 open NICU, where parents were always welcome but played a passive role. Kangaroo-mother (father)-care was systematically encouraged.

### Data collection and outcome definitions

Patient’s data were prospectively (FICare and CC) or retrospectively (HC) collected from the medical records. Among the perinatal-neonatal data intrauterine growth restriction, antenatal steroids, multiple birth, sex, gestational age, birth weight, 5 min Apgar score and the score for neonatal Acute Physiology Perinatal Extension (SNAPPE-II)^14^ were recorded. Main short-term neonatal outcomes included feeding practices (breastfeeding and day of life to full enteral feeding) and maturation skills (day of life to full oral feeding), weight gain, late-onset sepsis, necrotizing enterocolitis (NEC), stoma carrier, bronchopulmonary dysplasia (BPD), days on respiratory support, oxygen at discharge, term-equivalent age cranial ultrasound (cUS) diagnoses (normal, grade III intraventricular haemorrhage/periventricular haemorrhagic infarction, or white matter damage) in the preterm cohort or discharge normal cUS in the term cohort, severe retinopathy of prematurity (ROP), exitus, or length of hospital stay (NICU, intermediate care and total), and postmenstrual age at discharge. Finally the mid-term outcome recorded was the number of visits to the Emergency Services after 6 months from discharge. Small for gestational age was defined as a birth weight less than the 10th centile according to reference data from Fenton’s growth charts.^15^ Breastfeeding during admission was defined as having received their own mother’s milk at any time during admission (exclusive or mixed). Exclusive breastfeeding referred to patients who only received their own mother’s milk throughout admission. Direct breastfeeding indicated sucking directly at the breast. Full enteral nutrition was considered when reaching 130 ml/kg/day and parenteral nutrition or fluid therapy was halted. Late-onset sepsis was defined as a positive blood culture or antibiotics for 5 days regardless of microbiology, and NEC was considered in case of Bell’s modified stage ≥2.^16^ Severe ROP was defined if staging ≥3 or need for treatment.^17^ BPD was diagnosed on the basis of the need of supplemental oxygen at 36 weeks postmenstrual age.^18,19^ We only counted number of visits to the Emergency Service during the 6 months after discharge but not hospital readmissions.

### Statistical analysis

Qualitative data are expressed as absolute frequencies and percentages and the quantitative data using median and interquartile range. The normality of the continuous variables was studied using the Kolmogorov-Smirnov test. Given the absence of normality in the same variable in the different groups, median (IQR) were used to harmonize the results. For the association between categorical variables, the chi-square test or the Fisher test were used. To study the relationship between continuous and categorical variables the Mann–Whitney U test was used as non parametric test. Multivariate models were used to test for the potential association between FICare intervention and the infants’ outcomes, adjusted by variables that significantly differed between the three groups. All statistical tests were considered bilateral and as significant values, those p lower 0.05. The data were analysed with the statistical program SAS 9.3(SAS Institute, Cary, NC).

The power of the study has been calculated from the data of the sample size obtained and the percentage of change presented on breastfeeding rate at discharge (effect size). The PASS 15 Power Analysis and Sample Size Software (2017) program was used for this purpose.

## Results

### Patients

**Fig. 1:**
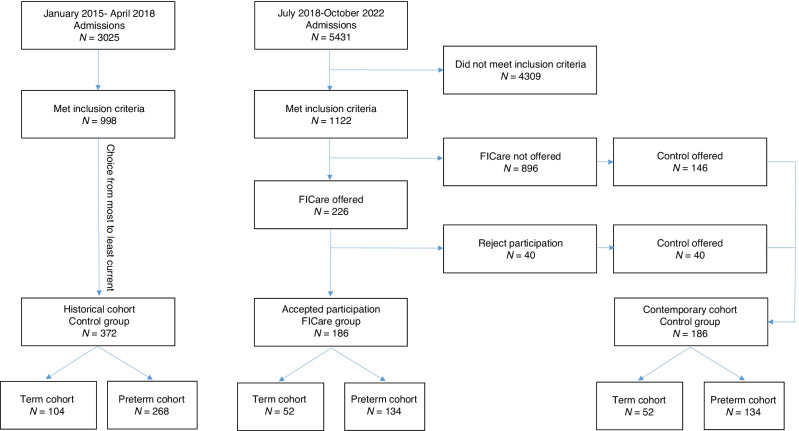
Study participants flow chart during the observation periods. The contemporary (FICare and Control cohorts)(July 2018-October 2022) and the historical cohort (January 2015-April 2018) are represented. The main reason for not offering FICare intervention to the contemporary controls was having exceeded the capacity established by protocol (13).

**Fig. 2:**
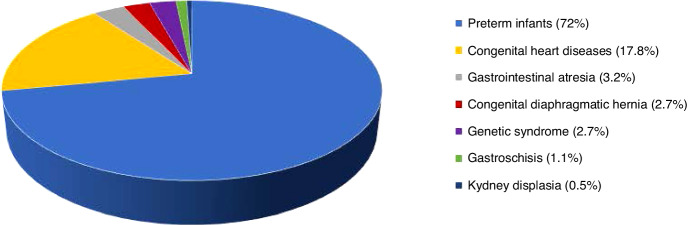
Distribution of main neonatal diagnoses among the study participants. All patients in the three cohorts are represented according to their predominant neonatal condition.

A total of 744 infants conform the study population; among them, 186 infants (and their families) received the intervention (FICare group) (Fig. 1), while 186 (CC) and 372 (HC) served as controls. Seventy two percent were born preterm and the remaining suffered complex neonatal conditions that deserved highly-specialized care (Fig. 2).

Groups were comparable with regards to the main clinical features on admission (Table 1). However, FICare group showed higher SNAPPE-II scores than the HC (preterm and term infants) and the CC (only for term infants). Preterm FICare infants also had lower 5 min Apgar scores than preterm HC infants.

**Table 1:** Clinical features of study participants

	FICare (*n* = 186)		Contemporary cohort (*n* = 186)		Historical cohort (*n* = 372)		FICare vs CC *p* value	FICare vs HC *p* value
	Preterm (*n* = 134)	Term (*n* = 52)	Preterm (*n* = 134)	Term (*n* = 52)	Preterm (*n* = 268)	Term (*n* = 104)	preterm/ term	preterm/ term
Male, *n* (%)	71 (53)	33 (63.5)	68 (51)	28 (53.8)	137 (51)	64 (61.5)	0.76/0.31	0.72/0.81
GA (weeks^days^), median (IQR)	28^6^ (26^5^–31^0^)	38^5^ (37^0^–39^5^)	28^6^ (26^6^–31^0^)	38^2^ (37^0^–39^5^)	28^6^ (26^4^–31^0^)	38^2^ (36^5^–39^4^)	0.88/0.70	0.93/0.47
Multiple births *n* (%)	53 (39.6)	1 (1.9)	43 (32.1)	0	105 (39.5)	11 (10.6)	0.20 / -	0.98/ -
BW (g), median (IQR)	1151 (855–1403)	3045 (2552-3310)	1105 (890–1349)	2915 (2450–3240)	1076 (813–1441)	2900 (2340–3180)	0.49/0.35	0.81/0.36
IUGR, *n* (%)	16 (12)	7 (13.5)	19 (14.5)	7 (15.2)	31 (11.7)	20 (23)	0.55/0.80	0.91/0.16
5’ Apgar score, median (IQR)	8 (6–9)	9 (8–9.5)	8 (6–9)	9 (8–10)	8 (7–9)	9 (8–10)	0.26/0.48	0.02/0.95
ANS, median (IQR)	2 (2–2)	-	2 (2–2)	-	2 (2–2)	-	-	-
SNAPPE-II, median (IQR)	10 (0–30)	5 (0–14)	10 (0–28)	0 (0–8)	0 (0–17)	0 (0–5)	0.33/0.01	0.00/0.01

The chi-square test or the Fisher test were used to study the association between categorical variables. The Mann–Whitney U test was used to study the relationship between continuous and categorical variables.

*CC* contemporary cohort, *HC* historical cohort, *GA* gestational age, *BW* birth weight, *IUGR* intrauterine growth restriction, *ANS* antenatal steroids (complete course), *SNAPPE-II* score for neonatal Acute Physiology Perinatal Extension^14^.

Median (IQR) postnatal age (days) at the start of the study intervention was 7.5 (6–14) and 8 (4.5–15) for the preterm and term groups, respectively.

### Preterm cohorts outcomes

Rates of breastfeeding during admission and at discharge were higher in the FICare group compared to the CC and the HC (Table 2). The rates of exclusive direct breastfeeding at discharge were also higher. These results remained after adjusting by 5’ Apgar score and SNAPPE-II (*p* < 0.01). No differences in growth patterns were found between the intervention and control groups.

**Table 2:** Main clinical outcomes of the preterm infant study participants

	FICare (*n* = 134)	Contemporary cohort (*n* = 134)	Historical cohort (*n* = 268)	FICare vs CC *p* value	FICare vs HC *p* value
Breastfeeding during admission, *n* (%)	126 (94.7)	112 (84.2)	206 (86.9)	0.01	0.02
Exclusive breastfeeding at discharge, *n* (%)	103 (79.8)	43 (37.7)	45 (20.6)	<0.01	<0.01
Exclusive direct breastfeeding at discharge, *n* (%)	25 (20.3)	3 (2.3)	7 (3.2)	<0.01	<0.01
ETF at discharge, *n* (%)	4 (3.1)	1 (0.8)	7 (3.2)	-	0.93
DOL to full enteral feeding, median (IQR)	10 (7–15)	9 (6–14)	10 (7–18)	0.72	0.45
DOL to full oral feeding, median (IQR)	52 (35–75)	45 (33–67)	46 (28.5–66.5)	0.12	0.08
Weight at discharge (g), median (IQR)	2530 (2182–2820)	2360 (2140–2805)	2400 (2180–2825)	0.06	0.30
Weight gain (g/day), median (IQR)	20.9 (18.9–24.2)	20.4 (18.5–23.3)	20.4 (18.2–23.4)	0.49	0.38
Late-onset sepsis, *n* (%)	48 (36)	50 (37.3)	122 (48.2)	0.83	0.02
NEC, *n* (%)	6 (4.5)	8 (6)	21 (8.2)	0.59	0.17
Stoma, *n* (%)	7 (5.3)	7 (5.2)	11 (4.3)	0.98	0.68
BPD, *n* (%)	44 (34.1)	25 (25.8)	73 (29.6)	0.18	0.36
Days on respiratory support, median (IQR)	33 (5–72.7)	23.5 (3–62.2)	22 (2–65)	0.04	0.04
Oxygen at discharge, *n* (%)	23 (17.8)	13 (10.2)	35 (14.3)	0.08	0.37
TEA normal cUS, *n*(%)	102 (77.3)	103 (79.8)	192 (77.4)	0.61	0.97
IVH grade III/PVHI, *n*(%)	21 (15.9)	18 (13.4)	29 (11.7)	0.22	0.24
WMI, *n*(%)	20 (15.2)	22 (17.1)	42 (16.9)	0.67	0.65
Severe ROP, *n* (%)	10 (7.8)	9 (7)	14 (5.7)	0.82	0.45
Exitus, *n* (%)	4 (3)	6 (4.7)	22 (8.2)	0.51	0.04
NICU length of stay (days), median (IQR)	29.5 (10–49.2)	16 (7.7–37.2)	18 (7–42)	0.02	0.01
IC length of stay (days), median (IQR)	32 (23–41)	36 (28–46)	35 (27–47)	0.02	0.02
Total length of stay (days), median (IQR)	63 (40.7–88)	56 (44.7–81.2)	57 (35–83)	0.31	0.15
PMA at discharge, median (IQR)	37^6^ (36^5^–39^4^)	37^3^ (36^2^–39^4^)	37^2^ (36^0^–39^1^)	0.25	0.27
Visits to Emergency Service ≤6 months from discharge, median (IQR)	0 (0–1)	1 (0–1)	1 (0–2)	<0.01	<0.01

The chi-square test or the Fisher test were used to study the association between categorical variables. The Mann–Whitney U test was used to study the relationship between continuous and categorical variables.

*CC* contemporary cohort, *HC* historical cohort, *ETF* enteral tube feeding, *DOL* day of life, *NEC* necrotizing enterocolitis ≥2 Bell’s modified stage, *BPD* bronchopulmonary dysplasia defined as oxygen dependency at 36 weeks post-menstrual age, *TEA* term equivalent age, *cUS* cranial ultrasound, *IVH* intraventricular haemorrhage, *PVHI* periventricular haemorrhagic infarction, *WMI* white matter injury, *ROP* retinopathy of prematurity, *IC* intermediate care, *PMA* postmenstrual age, *p* value, lack of cases.

Regarding other clinical outcomes, late-onset sepsis was less frequent in FICare compared to HC, while no differences were found between the former and the CC. Infants on FICare remained longer time on mechanical ventilation compared to the CC or HC, but need of supplemental oxygen at discharge was similar. Overall mortality was lower in the FICare group only respect to the HC.

No differences were found in total hospital length of stay; although length of NICU admission was longer in the FICare group compared to both control groups, intermediate care admission was shorter, and frequentation of Emergency Services after discharge lower in the FICare group compared to the CC and the HC.

Group sample sizes of 134 and 268 achieve 100,00% power to reject the null hypothesis of zero effect size when the population effect size is 1,27 and the significance level (alpha) is 0,050 using a two-sided z test.

The preterm CC and HC did not differ in any of the variables that were considered in these analyses.

### Term cohorts outcomes

Breastfeeding rates during admission and at discharge were higher in the intervention compared to both control groups (Table 3). Infants in FICare also showed higher rates of exclusive direct breastfeeding at discharge. These results were confirmed by multivariable analyses adjusted by 5’ Apgar score and SNAPPE-II (*p* < 0.01). Age to reach full oral feeding was earlier in FICare infants than in their CC (*p* < 0.06) and HC (p < 0.01) peers without differences in growth patterns between the study groups.

**Table 3:** Main clinical outcomes of the term infant study participants

	FICare (*n* = 52)	Contemporary cohort (*n* = 52)	Historical cohort (*n* = 104)	FICare vs CC *p* value	FICare vs HC *p* value
Breastfeeding during admission, *n* (%)	47 (90.4)	40 (78.5)	61 (58.7)	0.01	<0.01
Exclusive breastfeeding at discharge, *n* (%)	34 (85)	12 (37.5)	11 (12.9)	<0.01	<0.01
Exclusive direct breastfeeding at discharge, *n* (%)	19 (40.4)	6 (14.6)	2 (2.4)	0.01	<0.01
ETF at discharge, *n* (%)	10 (21.3)	13 (28.3)	19 (20.4)	0.43	0.90
DOL to full enteral feeding, median (IQR)	16.5 (10.5–21.7)	19 (8–30.75)	20 (17.7–31)	0.26	0.33
DOL to full oral feeding, median (IQR)	24 (17.7–37)	34 (23–50)	33 (23–47)	0.06	0.01
Weight gain (g/day), median (IQR)	14.7 (8.1–17.8)	14.6 (12.3–20)	14 (9.2–18.3)	0.70	0.64
Late-onset sepsis, *n* (%)	17 (33.3)	27 (51.9)	60 (58.8)	0.06	0.03
NEC, *n* (%)	1 (1.9)	6 (11.5)	8 (7.8)	-	-
ECMO, *n* (%)	9 (17.3)	3 (5.9)	7 (6.8)	0.04	0.04
Days on respiratory support, median (IQR)	16.5 (9-32.2)	17 (9–34)	18 (7–43.2)	0.86	0.89
Oxygen at discharge, *n* (%)	4 (8.2)	5 (10.9)	11 (11.1)	0.65	0.57
Discharge normal cUS, *n*(%)	44 (88)	42 (83.3)	77 (77.8)	0.79	0.13
Exitus, *n*(%)	6 (11.5)	5 (9.6)	5 (4.8)	0.75	0.61
NICU length of stay (days), median (IQR)	25.5 (19–34.7)	27.5 (19–38)	24 (16–37)	0.64	0.44
IC length of stay (days), median (IQR)	10 (5.7–19)	24 (13–48)	20 (15–32)	<0.01	<0.01
Total length of stay (days), median (IQR)	35 (27–62)	51.5 (35–89.5)	45 (32–69)	0.01	0.03
PMA at discharge, median (IQR)	44^0^(41^4^–46^6^)	46^3^(43^3^–50^1^)	45^1^(42^4^–47^5^)	0.01	0.21
Visits to Emergency Service ≤6 months from discharge, median (IQR)	0 (0–0.75)	1 (0–2.2)	1 (0–3)	<0.01	<0.01

The chi-square test or the Fisher test were used to study the association between categorical variables. The Mann–Whitney U test was used to study the relationship between continuous and categorical variables.

*CC* contemporary cohort, *HC* historical cohort, *ETF* enteral tube feeding, *DOL* day of life, *NEC* necrotizing enterocolitis ≥2 Bell’s modified stage, *ECMO* extracorporeal membrane oxygenator, *TEA* term equivalent age, *cUS* cranial ultrasound, *IC* intermediate care, *PMA* postmenstrual age, p value, lack of cases.

Late-onset sepsis was lower in FICare than in CC (*p* = 0.06) and HC (*p* = 0.03) groups. Although more infants in the FICare needed ECMO, no differences in other co-morbidities or overall mortality were found between the intervention and control groups (Table 3). Intermediate care and total length of stay were shorter in FICare than in controls (CC and HC), and discharged occurred at an earlier post-menstrual age in the former. Number of visits to Emergency Services within the first 6 months after discharge were lower in FICare compared to controls.

Group sample sizes of 52 and 104 achieve 100,00% power to reject the null hypothesis of zero effect size when the population effect size is 1.02 and the significance level (alpha) is 0.050 using a two-sided z test.

The term CC and HC infants did not differ in any of the variables that were considered in these analyses.

## Discussion

This is the first study carried out at a tertiary NICU attending all type of complex conditions, either medical or surgical, where the benefits of FICare in the short and mid-term outcomes of critically ill term infants are reported; and supports previous intervention-related benefits reported in the preterm infant.^1–3,5,6,8,9^ Our study cohorts differ from those included in most studies about FICare in two features. First, our study gathers a wider variety of main neonatal diagnoses as well as gestational age ranges. Second, the complexity of FICare intervention is higher due to a wider family caregiver curricula and an earlier infant-family dyad enrolment and start of intervention.^13^ Of note, a recent but rather small study reports on the feasibility of FICare implementation in a larger and sicker population of newborns compared to their own standards.^19^

Both, preterm and term infants cared according to FICare policies had higher rates of breastfeeding during admission and at discharge and showed better maturation skills, supported by the higher rates of direct breastfeeding at discharge, than those under standard care. Although these positive effects of FICare on breastfeeding rates at discharge have been already reported,^1,2,7,8^ most studies refer to either mixed breastfeeding or predominantly breastfeeding^1,7,8^ but not to exclusive breastfeeding. In addition, length of stay in intermediate care facilities was shorter in FICare infants, with a reduction of 3–4 days in preterm and 10–14 days in term infants; and total length of stay was shorter in term infants in FICare compared to those in usual care. The reported reduction in length of stay with FICare, without specifying the type of specialized care provided, ranges between 2.5 and 14 days.^3,5,6,9^ The fact that we did not find an intervention-related reduction in NICU stay is probably explained on the basis of a poorer condition at birth and more severe disease state of either term or preterm infants in our FICare cohorts. Five min Apgar score was lower in preterm FICare compared to HC peers; SNAPPE-II scores were higher in term FICare compared to both CC and HC, and also in preterm FICare compared to HC; and duration of mechanical ventilation was longer in preterm FICare compared to their CC and HC peers. These results are particularly striking in the term infant group where the higher disease severity is clearly highlighted by the fact of higher rates of ECMO treatment in FICare compared to CC or HC. This is of utmost relevance because mortality rates are not different (term infants) or even lower (preterm infants).

Our data also point towards a positive effect on family’s empowerment. In fact, Emergency Services frequentation within the first 6 months after discharge was lower in both FICare groups, i.e., the preterm and term cohorts, which at the best of our knowledge is reported for the first time as a FICare-related goodness. Decreased parental anxiety and post-traumatic stress is probably behind this effect, although has not been systematically assessed in this study by the appropriate validated tests.

We have also observed a reduction in late-onset sepsis as previously described.^3,4,8^ However, we only were able to demonstrate this finding when FICare group was compared with the HC. Two things may explain this finding; first, the implementation of an action plan at a national level during 2016, the so called *“Bacteriemia Zero”*, that gathered a package of measures against nosocomial infection, promoted and launched through a multidisciplinary team; second, an effect of contamination of the study intervention in the CC, because actions related to hand hygiene were probably more easily widespread also in this cohort.

It is also worth highlighting the lower rates of NEC in the intervention group compared to the CC and HC of term infants, although the scarcity of cases prevented to demonstrate any statistical significance. This potential FICare benefit should be confirmed in larger study populations.

We think that the positive trends observed during the intervention period on breastfeeding patterns are not related to actions other than FICare. In fact, policies that may directly impact breastfeeding rates, such as availability of human milk bank or lactation consultant, were operational in the case of the former since 2014 and in the latter from September 2022, that means, long time before the intervention started or at the end of the study period.

Although a cost-effectiveness analysis is beyond the scope of this study, according to the trends observed on hospital length of stay and emergency visits post-discharge, we can indeed remark a positive effect on reduced hospital resources and spared money.

Our study has several limitations. First, although we have gathered a large population for analyses, ours is not a randomized clinical trial. The nature of the intervention makes for an unfeasible setting for blinding, so that contamination would have been always an issue. Second, it is a single center trial, with our own particularities in terms of type of patients attended and procedures, rising concerns about generalization. Third, as this was a pilot study to assess FICare implementation at our site, patient enrolment could have been biased. For instance, the number of patient-family dyads available for enrolment at a given time point was limited so that the corresponding peer in the CC might not had been invited to participate because the quota was full instead of refusal to enter the FICare program. Finally, we were not able to analyse the psychological impact of the model in families due to the low rate of completed questionnaires by participants.

Our study has several strengths. Overall, the inclusion of critically ill term infants in addition to the unstable premature. This fact really confirms that scalability of the FICare model is feasible even in a complex context where patients are usually cared by a multidisciplinary team, surgeons among others. The inclusion of a HC is another key point that permitted to counteract the eventual effect of intervention contamination within the CC. Third, the co-creation process to adapting and implementing FICare at our site was accomplished through the collaboration of healthcare professionals from different grounds and levels, together with veteran parents and associations. This was critical for the program’s success as the needs, expectations and possibilities were really considered a priority and prosecuted. The continuous evaluation of the progress of FICare implementation, and the adjustments accomplished during the ongoing process, have permitted us to generalize the method to all our customers that currently want it. Finally, the piloting of FICare model was carried out at a time when our NICU was an open-bay unit, which represents not only an added challenge from an architectural point of view, but also from the perspective of privacy and organization. Nevertheless, the benefits reported in our study bring additional support for a wider use of the method regardless architectural barriers. So far, we understand that our recent experience adds valuable information to support FICare generalization as the new NICU standard of care.

## Conclusions

This is the first report on the short and mid-term impact of the FICare model in a broader population of unstable, sick neonates cared for in an open-bay NICU. The benefits found on parental empowerment and infants’ health outcomes, applying not only to the stable preterm infant but particularly to the term cohort that suffered a variety of complex diseases deserving multidisciplinary care, support the scalability of FICare model to the more complex healthcare neonatal services.

## Data Availability

All data generated or analysed during this study are included in this published article.
